# Actionable Driver Events in Small Cell Lung Cancer

**DOI:** 10.3390/ijms25010105

**Published:** 2023-12-20

**Authors:** Mirian Gutiérrez, Irene Zamora, Michael R. Freeman, Ignacio J. Encío, Mirja Rotinen

**Affiliations:** 1 Department of Health Sciences, Public University of Navarre, 31008 Pamplona, Spain; mirian.gutierrez@unavarra.es (M.G.); zamora.127247@e.unavarra.es (I.Z.); 2 Departments of Urology and Biomedical Sciences, Cedars-Sinai Medical Center, Los Angeles, CA 90048, USA; michael.freeman@cshs.org; 3 Department of Medicine, University of California Los Angeles, Los Angeles, CA 90095, USA; 4 IdiSNA, Navarre Institute for Health Research, 31006 Pamplona, Spain

**Keywords:** small cell lung cancer, heterogeneity, targeted therapy, inhibitor molecules, clinical vulnerability

## Abstract

Small cell lung cancer (SCLC) stands out as the most aggressive form of lung cancer, characterized by an extremely high proliferation rate and a very poor prognosis, with a 5-year survival rate that falls below 7%. Approximately two-thirds of patients receive their diagnosis when the disease has already reached a metastatic or extensive stage, leaving chemotherapy as the remaining first-line treatment option. Other than the recent advances in immunotherapy, which have shown moderate results, SCLC patients cannot yet benefit from any approved targeted therapy, meaning that this cancer remains treated as a uniform entity, disregarding intra- or inter-tumoral heterogeneity. Continuous efforts and technological improvements have enabled the identification of new potential targets that could be used to implement novel therapeutic strategies. In this review, we provide an overview of the most recent approaches for SCLC treatment, providing an extensive compilation of the targeted therapies that are currently under clinical evaluation and inhibitor molecules with promising results in vitro and in vivo.

## 1. Introduction

Lung cancer is the leading cause of cancer death, with 1.8 million estimated deaths and over 2 million cases diagnosed worldwide in 2020 [1,2]. The disease is subdivided into two fundamental histological groups: non-small cell lung cancer (NSCLC) and small cell lung cancer (SCLC). This classification is based on the microscopic characteristics of the tumor cells and is crucial for determining the appropriate therapeutic approach. SCLC makes up about 15% of lung cancer cases and is the most aggressive subtype, characterized by an exceptionally high proliferation rate and significant metastatic potential, leading to a very poor prognosis. Despite the initial responsiveness to chemotherapy, the majority of patients with SCLC will eventually relapse, with a dismal 5-year survival of less than 7% and most patients dying within 12 months [3]. The etymology of SCLC is strongly associated with tobacco, with smokers representing 98% of patients [4]. The average patient is a male over 70 years of age with significant current previous tobacco exposure and pre-existing pulmonary, cardiovascular, and metabolic conditions [5].

At present, chemotherapy is the primary approach to treatment of SCLC. According to the ESMO guidelines, surgery is considered for patients diagnosed at stages I or II, and is typically combined with chemoradiotherapy. For cases with complete resection and limited or no nodal involvement, cisplatin-etoposide is recommended. However, two-thirds of patients are diagnosed at the metastatic or extensive stage disease [3]. In these situations where resection is not possible, chemotherapy takes precedence as the first-line treatment. Irrespective of the disease stage at diagnosis, the guidelines advise prophylactic brain irradiation to mitigate the risk of brain metastasis [6]. 

Despite the initial favorable responses to chemotherapy, relapses are frequent after the first year of treatment [7]. Over the past few decades, second-line treatments such as cyclophosphamide/doxorubicin/vincristine (CAV), topotecan, or lurbinectedin have been approved. Immunotherapy is gaining attention due to its potential to suppress immunologically active tumors, and may be particularly applicable in SCLC due to its high immunogenicity, which promotes expression of neoantigens and their subsequent recognition by the immune system [6,8,9]. Currently, immunotherapy is used as monotherapy as a third-line strategy, and many trials are exploring the possibility of its application as a first-line therapy in combination with chemotherapy [6]. The recent IMpower133 and CASPIAN clinical trials have provided compelling support for the immune checkpoint inhibitors (ICIs) atezolizumab and durvalumab in combination with chemotherapy. This approach has demonstrated significant enhancements in both overall survival (OS) and progression-free survival (PFS) when compared with chemotherapy alone in the context of extensive stage disease [10,11]. However, in SCLC the effectiveness of immunotherapy is lower than in other solid tumors such as NSCLC due to the variety of mechanisms employed by these tumors to evade the immune system, and only a small percentage of patients demonstrate lasting responses [3].

In an era where recent advances in personalized medicine have enabled the development of targeted therapies tailored to the molecular alteration characteristic of each condition, SCLC continues to be managed as a single entity. However, there are many ongoing efforts for the discovery of new targets and their therapeutic application. This review compiles the most recent studies that have successfully translated findings into clinical trials or may do so in the near future.

### 1.1. Mutational Profile and Molecular Landscape of SCLC

SCLC is a genetically intricate condition characterized by a high mutation rate of approximately nine non-synonymous mutations per mega-base. Its mutational profile is highly associated with tobacco carcinogens, and most patients present dual loss of the tumor suppressors p53 and RB [12]. Their functional paralogs TP73 and RBL2 (RB Transcriptional Corepressor Like 2), respectively, are mutated in a significant portion of SCLC patients as well [13].

Among other common events in this type of cancer are mutations in genes that encode enzymes modifying histones, such as the histone acetyltransferases CREBBP (CREB Binding Protein) and EP300 (E1A Binding Protein P300), as well as the histone methyltransferases MLL (Mixed Lineage Leukemia). Genes encoding chromatin modifying complexes (e.g., BAF-SWI/SNF and PBAF-SWI/SNF complexes) have been identified as being frequently mutated in SCLC, including the ARID1A/B (AT-rich Interaction Domain 1A and 1B), BRG1 (or SMARCA4), PBRM1 (Polybromo-1), and CHD7 (Chromodomain Helicase DNA Binding Protein 7) genes [14]. SCLC tumors show recurrent somatic alterations in proteins related to cytoskeleton formation or cell–cell and cell–matrix interactions, such as SLIT2 (Slit Guidance Ligand 2) and mutagenic alterations in kinase receptors, like the inactivation of EPHA7 (EPH Receptor A7) and/or the amplification of FGFR (Fibroblast Growth Factor Receptor) and MET [15]. The PI3K (Phosphatidylinositol 3-kinase)/AKT/mTOR (Mammalian Target of Rapamycin) signaling pathway, linked to enhanced proliferation and migration of SCLC cells, is another frequently altered pathway, with activating mutations found in PIK3CA (Phosphatidylinositol-4,5-Bisphosphate 3-Kinase Catalytic Subunit Alpha), AKT, and PTEN (Phosphatase and Tensin Homolog) genes [16].

**Figure 1:**
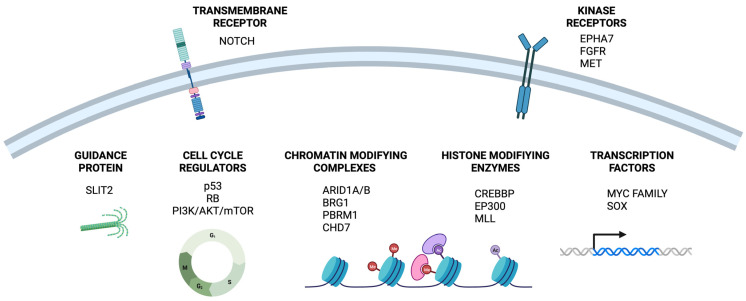
Representation of molecular alterations that play a role in SCLC progression and recurrence.

Inactivating mutations in NOTCH family members, especially NOTCH1, are present in 25% of SCLC tumors [12]. In the context of RB1 and p53 loss, loss of Notch drives SCLC cells into a stem-like state, thereby contributing to cancer progression. Nevertheless, Notch is active in a subset of human SCLC, and this pathway has been described as playing a key role in directing tumor transdifferentiation towards a non-neuroendocrine state, thereby promoting drug resistance mechanisms [17]. The MYC family of transcription factors (C-MYC, L-MYC and N-MYC) exhibit mutually exclusive amplifications in 20% of SCLC tumors [15]. C-MYC expression and activity is associated with increased tumor progression and metastatic potential, and a large body of evidence suggests that it is involved in the reverse neuroendocrine transdifferentiation (NET) process [17]. The SOX family of transcription factors, particularly SOX2 (SRY-Box Transcription Factor 2), is frequently amplified and overexpressed in SCLC tumors and cell lines. Upregulation of SOX2 is regarded as a pivotal event in SCLC, where the protein functions as a lineage-survival oncogene that contributes to the transcriptional deregulation observed in the disease and promotes the classical neuroendocrine phenotype [13,18]. The whole set of molecular alterations described above is depicted in Figure 1. 

### 1.2. Tumor Heterogeneity and Plasticity in SCLC

SCLC is a high-grade malignant epithelial tumor. The pulmonary epithelium contains various populations of stem and progenitor cells that give rise to the variety of differentiated cell types in the lung. The fact that SCLC cells express neuroendocrine markers has led to the assumption that they originate from neuroendocrine cells in the pulmonary epithelium or their neuroendocrine progenitors [19]. Supporting this theory, these cells express neuroendocrine transcription factors such as ASCL1 (Achaete-Scute Homolog 1), which plays a crucial role in the proper development of pulmonary neuroendocrine cells and has been shown to be essential for the survival of SCLC cells [20]. However, it has been demonstrated that deleting p53 and RB1 in distinct lung cell types can give rise to different SCLC subtypes, implying that the cell of origin can potentially define the trajectory of tumor progression [21,22]. In addition to a de novo origin, SCLC can arise as a result of histological transformation in response to adverse conditions, such as hypoxia or selective pressure to escape targeted therapy against an oncogenic driver in lung adenocarcinoma. This plasticity, referred to as NET, has been identified in more than 14% of patients with EGFR (Epidermal Growth Factor Receptor)-mutated NSCLC in response to resistance to EGFR inhibitor therapy [23,24].

While the majority of SCLC tumors and cell lines display a morphology in concordance with the ‘classic’ WHO description of SCLC, and express a comprehensive range of neuroendocrine markers, there is a subgroup of variants characterized by reduced or absent expression of these markers coupled with changes in morphology and growth potential [25,26]. The transcriptomic analysis of both these cell lines and SCLC tumors has enabled the categorization of SCLC into four molecular subtypes, determined by the expression of a range of transcription factors that specify cell lineage: ASCL1, NEUROD1 (Neuronal Differentiation 1), POU2F3 (POU Class 2 Homeobox 3), or YAP1 (Yes1 Associated Transcriptional Regulator). The distinctions among the different transcriptional profiles of these subtypes involve different levels of neuroendocrine differentiation and disparities in their metabolic traits [3].

The ASCL1 subtype (SCLC-A), which comprises about 70% of SCLC cases, is characterized by predominant expression of ASCL1, a transcription factor critical for the normal development of pulmonary neuroendocrine cells (PNECs) [17]. ASCL1 also plays a vital role in the growth and survival of in vitro cell lines and the development of high-grade neuroendocrine lung tumors in murine models [27]. ASCL1 triggers the expression of oncogenes such as L-MYC, RET, SOX2, and NFIB (Nuclear Factor I B), in addition to other anti-apoptotic genes such as BCL2 (B-cell Lymphoma 2) and genes responsible for the neuroendocrine phenotype [28]. Importantly, the Notch signaling pathway negatively regulates ASCL1 expression, establishing an antagonistic relationship between the activation of the Notch signaling pathway and ASCL1 expression [29].

NEUROD1 also plays a crucial role in development of PNECs, and is the main driving transcription factor for 17% of SCLC tumors [28]. Note that there is a subset of tumors with varying levels of dual expression of ASCL1 and NEUROD1, usually with a predominance of ASCL1 expression and activity. The NEUROD1 subtype (SCLC-N) shows high expression of neuroendocrine markers, although to a lesser extent than SCLC-A [28]. NEUROD1 significantly contributes to the promotion of malignant traits, enhancing the survival and migratory abilities of tumor cells, and activates oncogenes such as C-MYC [27].

The YAP1 subtype (SCLC-Y), accounting for approximately 7% of SCLC tumors, is characterized by predominant expression of YAP1, a critical mediator in the activation of the Hippo signaling pathway. The existence of this subtype has sparked controversy, as several groups have failed to identify tumors exclusively expressing YAP1 [30,31,32]. Its association with an inflammatory phenotype has led to the proposal of the SCLC-I (inflamed) subtype to replace SCLC-Y. This subtype exhibits the highest levels of immune cell infiltration into the tumor, and is identified by a mesenchymal phenotype and a non-neuroendocrine profile with low or absent expression of ASCL1 and NEUROD1 [33,34].

The POU2F3 subtype (SCLC-P), representing 7% of SCLC tumors, is characterized by the absence of expression of ASCL1, NEUROD1, and neuroendocrine markers [28]. POU2F3 is a transcription factor essential for the differentiation of tuft cells, a rare type of chemosensory cells found in the pulmonary epithelium. These cells, similar to neuroendocrine cells, respond to external stimuli by releasing bioactive substances that regulate the functions of local immune and epithelial cells [35]. This subtype is rarely found in tumors originating from PNECs [28]. Similar to the SCLC-Y subtype, it exhibits a non-neuroendocrine phenotype and has been associated with an inflammatory phenotype [30].

**Figure 2:**
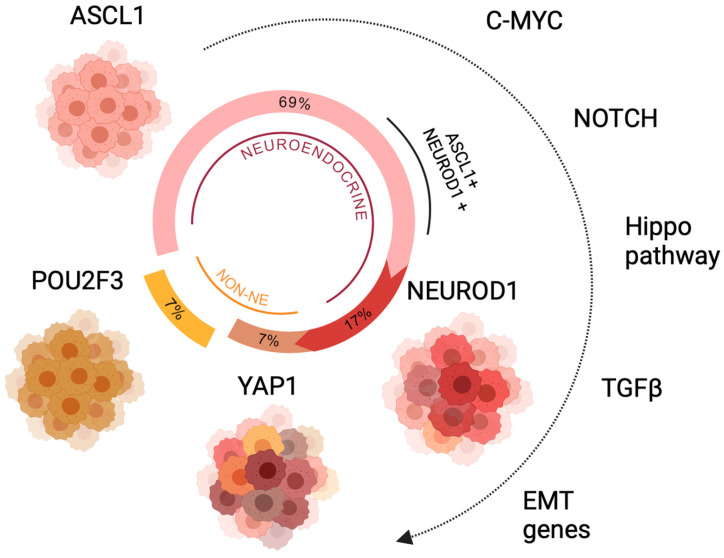
Scheme of dynamic evolution across the different subtypes of SCLC, showing the cell plasticity drivers and frequency of occurrence for each molecular subtype or cell state. EMT: epithelial-to-mesenchymal transition.

Recently, it has been demonstrated that these subpopulations can coexist within the same tumor as a result of lineage plasticity, with the ASCL1 subtype evolving into the NEUROD1 subtype and the latter into the YAP1 subtype [17,36], implying that these phenotypes are different phases within the same evolutionary process rather than distinct subtypes. This transformation from a neuroendocrine to a non-neuroendocrine state appears to be orchestrated by a set of critical genes and cellular programs such as the previously stated C-MYC (which activates Notch), Hippo, and TGFβ (Transforming Growth Factor β) signaling pathways as well as genes involved in the epithelial-to-mesenchymal transition (EMT) [17,26,37] (Figure 2). This continuum of plastic cell states illustrates the capacity of SCLC to trade off cancer hallmark-related tasks, enabling the cells to adapt to different microenvironments. This transforming capacity has been hypothesized to derive from the dysregulation of the innate plasticity of normal PNECs, which have been shown to differentiate in response to lung injury [38,39]. Other factors that may induce cell plasticity include tumor treatment and changes in microenvironment [38].

Plasticity should be considered an important target for SCLC treatment. The presence of multiple cellular subpopulations and their interactions in SCLC could explain the ability of these cells to acquire chemoresistance and high metastatic potential. The high degree of heterogeneity in SCLC highlights the need to develop new precision therapies specific to each subtype, as they present different degrees of neuroendocrine differentiation and distinctive alterations that imply different therapeutic vulnerabilities [40,41].

## 2. Actionable Drivers in SCLC

### 2.1. Targeting Transcription Factors and Epigenomic Regulators

LSD1 (also known as KDM1A) and KDM5A are lysine-specific histone demethylases involved in the regulation of the Notch signaling pathway. LSD1 is overexpressed in SCLC [42]. Iadademstat (ORY-1001) is an LSD1 inhibitor in phase 2 clinical trials in leukemia (NCT05546580) that has been shown to reactivate Notch signaling, thereby suppressing ASCL1 activity and reducing tumor growth in SCLC mouse models [43]. Another LSD1 targeted drug, GSK2879552, has been tested in SCLC in combination with a PD-1 inhibitor in a syngeneic model of SCLC, resulting in strong tumor growth inhibition [44]. However, the clinical trial conducted with GSK2879552 was terminated, as the risk–benefit assessment did not favor continuation [45]. KDM5A represses Notch signaling to sustain neuroendocrine differentiation and promote tumorigenesis. Concomitant inhibition of KDM5A and LSD1 with KDM5-C70 and ORY-1001 molecules, respectively, has been shown to synergistically repress ASCL1 and suppress proliferation of SCLC cell lines [46].

Another epigenetic factor that has emerged as a significant target in SCLC is EZH2 (Enhancer of Zeste Homolog 2), the enzymatic subunit of the Polycomb Repressive Complex 2 (PRC2). This histone methyltransferase is frequently overexpressed in this cancer type, and promotes SCLC progression by suppressing the TGFβ-Smad-ASCL1 pathway [47]. Various EZH2 inhibitors are under investigation for SCLC and other tumor types. A number of these inhibitors have progressed to clinical trials in SCLC, such as DS-3201b (NCT03879798), PF-06821497 (NCT03460977), and XNW5004 (NCT06022757). These molecules may be relevant in SCLC, as evidence suggests that they enhance standard cytotoxic therapy and prevent the development of resistance both in vitro and in vivo [48].

As previously mentioned, C-MYC activates Notch, and MYC family genes are amplified and overexpressed in SCLC [46]. The lack of an accessible enzymatic active site in this protein has entailed a challenge in the search for MYC targeting strategies [49]. A dominant-negative form that sequesters MYC away from DNA has been developed, exhibiting anti-tumoral efficacy in SCLC in vitro and in NSCLC models in vivo [50,51]. Other C-MYC repressors, such as the BET (Bromodomain and Extra-Terminal Domain) inhibitor JQ1, have shown good results in different cancer types, and could be tested in SCLC patients that express high C-MYC levels [52,53]. C-MYC has not only been studied as a target, but also as a biomarker. C-MYC-driven variants are vulnerable to Aurora kinase inhibition [54] and Chk1 (Checkpoint Kinase 1) inhibitors [55]. Considering that C-MYC amplification is characteristic of NEUROD1-positive tumors, therapies targeting C-MYC would be of most benefit in these patients [56].

The transcription factor CREB imparts neuroendocrine features and has been found to be elevated in SCLC tumors. CREB activity is related to cell proliferation, and its blockade with PKA inhibitors abolished the development of SCLC in an in vivo model [57]. As mentioned above, the acetyltransferase CREBBP is a frequently mutated gene in SCLC and its inactivation is associated with the SCLC-A subtype. Jia et al. demonstrated that knocking out CREBBP reduces transcription of cellular adhesion genes and drives tumorigenesis [58]. These effects could be restored with HDAC inhibitors such as pracinostat or tinostamustine, breaking new ground in the treatment of ASCL1-positive tumors [59].

ELF3 (E74-Like Factor 3) is an ETS transcription factor that exhibits context-dependent functionality, serving both as a lineage-dependent oncogene and as a tumor-suppressive gene [60,61]. In SCLC, ELF3 acts as an oncogenic regulator in ASCL1-positive cells. Because AURKA (Aurora Kinase A) is an ELF3 target gene, the use of AURKA inhibitors has been proposed for patients with high ELF3 expression [33]. Upstream inhibition of this factor using auranofin (a PKC inhibitor) leads to cell death in NSCLC [62]; thus, its use could be a potential therapy for patients with high levels of ELF3 in SCLC. Recently, the transcription factors ETV4 (ETS Variant Transcription Factor 4) and ETV5 (ETS Variant Transcription Factor 5) have been identified as key mediators of SCLC progression. Considering that both are downstream transcriptional effectors of FGFR (Fibroblast Growth Factor Receptor) signaling, the pan-FGFR inhibitor LY2874455 has emerged as a potential therapeutic candidate for SCLC treatment. LY2874455 not only decreases ETV4 and ETV5 protein expression, it blocks the downstream MAPK and PI3K-AKT signaling pathways. In pre-clinical studies, LY2874455 has demonstrated efficacy as an anti-tumor agent when used alone or in combination with cisplatin-etoposide. These findings highlight the potential of LY2874455 as a promising therapeutic approach for SCLC [63].

The signal transducer and activator of transcription 3 (STAT3) is often activated in SCLC; this is partly attributed to tumor-associated macrophages (TAMs). Consequently, the crosstalk between SCLC cells and TAMs is being considered as a potential target for SCLC treatment. Onionin A, a natural compound isolated from onion, has shown the ability to counteract the influence of macrophage-derived soluble factors, including IL-6, resulting in effective suppression of the SCLC cell proliferation both in vitro and in vivo [64].

### 2.2. Targeting DNA Damage Response Proteins

Lurbinectedin, aforementioned as one of the second-line therapies, is a synthetic marine-derived anticancer agent that acts as a selective inhibitor of oncogenic transcription [65]. This drug binds covalently to GC-rich regions and blocks the DNA repair mechanism, resulting in RNA Pol II elongation arrest and degradation [66]. Lurbinectedin selectively targets the CpG islands located downstream of the transcriptional start site of ASCL1 and NEUROD1, leading to suppression of these genes and their targets, including BCL2, INSM1 (Insulinoma-associated 1), C-MYC, and AURKA [67]. This drug was approved by the FDA for treatment of patients with metastatic SCLC after showing favorable results in a phase 2 basket trial of relapsed SCLC patients [68,69]. Although the phase 3 ATLANTIS trial demonstrated that a combination of lurbinectedin and doxorubicin did not improve OS compared to control therapy, this combination shows a safer hematological profile [65].

The high mutational burden of SCLC makes the DNA damage repair (DDR) pathway an attractive target for tumor control. The DNA repair protein PARP1 (Poly (ADP Ribose) Polymerase 1) was identified as a target in SCLC in a proteomic analysis [70]. This seminal study showed that PARP expression is higher in both SCLC cells and patients compared to NSCLC and demonstrated the high sensitivity of many SCLC cell lines to treatment with PARP inhibitors (PARPi), although sensitivity to these drugs was not universal for all cell lines tested [70,71]. The PARPi veliparib has been shown to be mostly ineffective when used as single-agent therapy; however, it potentiates standard chemotherapy in SCLC patients [72]. Veliparib combined with temozolomide, an oral alkylating agent that induces O^6^-alkyl-guanine lesions on DNA, has shown significant improvement in these patients, especially in those with SLFN11 (Schlafen 11)-expressing tumors [73]. SLFN11 is a putative DNA/RNA helicase that induces irreversible replication blockade. The ongoing NCT04334941 clinical trial aims to test the efficacy of the PARPi talazoparib plus immunotherapy based on SLFN11 status. SLFN11 serves as a predictive biomarker in a wide range of cancer therapies that cause DNA damage, including platinum salts, topoisomerase I/II inhibitors, and lurbinectedin [74,75]. The epigenetic inactivation of SLFN11-mediated resistance to DNA-targeted agents in different cancers has been bypassed with class I HDAC (Histone DeACetylases) inhibitors [76]. Other PARPis, such as olaparib and talazoparib (BMN-673), have been evaluated in SCLC as well [77,78]. 

Another potential target is the primary activator of the replication stress response, ATR (Ataxia Telangiectasia and Rad3-related). Its inhibitor, M6620 (berzosertib), has been shown to be effective in pre-clinical studies when used in combination with other SCLC therapies [79]. In combination with topotecan, it has a cytotoxic effect and activates the STING (STimulator of INterferon Genes) pathway, implying that ATR-TOP1 inhibition may improve response to immune checkpoint therapy in STING-low SCLC tumors [80]. In addition, synergy of M6620 with lurbinectedin has been demonstrated in multiple SCLC cell lines, organoids, and in vivo models [81], and their combined effectiveness is currently being assessed in a clinical trial (NCT04802174). Finally, other M6620 combinations are under investigation in SCLC, such as a combination with the Trop2 inhibitor molecule sacituzumab-govitecan (NCT04826341), a transmembrane glycoprotein that has emerged as a promising molecular target for lung cancer treatment [82].

### 2.3. Kinase Inhibitors

Continuing with the DDR pathway, certain kinases have emerged as compelling targets for SCLC. Indeed, one of the most studied family of molecules for SCLC treatment is the Aurora family of serine/threonine kinases (AURK). These enzymes are frequently found to be overexpressed in cancer, and play a key role in the regulation of mitosis and chromosome alignment [83]. It has been shown that MYC family-driven cell lines, in particular the NEUROD1 high subtype with C-MYC amplification or overexpression, are sensitive to Aurora K inhibitors [54,84]. Barasertib (AZD1152), an AURKB inhibitor, has shown efficacy suppressing growth of a subset of SCLC cell lines both in vitro and in vivo [85]. An extensive safety evaluation of this molecule and its derivatives has been conducted in clinical trials for advanced solid tumors, including patients with SCLC [86]. Additionally, Alisertib (MLN8237), a selective inhibitor of AURKA, has demonstrated improvement in PFS when combined with paclitaxel compared to paclitaxel alone in patients with SCLC tumors positive for C-MYC [87]. As a caveat, Aurora K inhibitor effectiveness and safety must be carefully validated in phase 3 clinical trials, as many patients, despite showing an initial good response, have had to discontinue treatment due to tumor progression or adverse effects, particularly those linked to blood malignancies [88].

As mentioned before, after DNA damage and in the absence of p53 suppressor activity, the ATR kinase activates the cell-cycle checkpoint kinase Chk1, acting as a principal mediator of cell cycle arrest in the G2/M phase [89]. Chk1 is highly expressed in SCLC lines compared to NSCLC [55]. Another study has shown that Chk1 expression is directly correlated with poor OS among SCLC patients [90]. In pre-clinical SCLC models, the Chk1 inhibitors prexasertib (LY2606368) and AZD7762 sensitize cells resistant to cisplatin [91]. Sen et al. demonstrated that the effectiveness of Chk1 inhibition using prexasertib was particularly pronounced in cells with C-MYC amplification or overexpression [55]. Although prexasertib displays strong efficacy in pre-clinical studies as a single agent, in clinical trials it did not achieve sufficient activity and exhibited limitations in terms of bioavailability [92]. As a result, novel Chk1 inhibitors such as SRA-737 are currently being tested in patients with advanced solid tumors, including SCLC [93]. Of note, it has been observed that targeting Chk1 in combination with chemotherapy enhances the effect of PD-L1 blockade by modulating the immune microenvironment, providing compelling rationale for combining Chk1 inhibitors with chemotherapy and ICIs [94].

Another regulator of the G2/M checkpoint, preventing the initiation of mitosis in the presence of DNA damage, is the evolutionarily highly conserved kinase WEE1. This kinase has become a target of interest in SCLC, and the first-in-class WEE1 inhibitor adavosertib (AZD1775) has demonstrated a good safety and tolerability profile in a phase 1 study conducted in patients with advanced solid tumors including SCLC [95]. WEE1 expression levels are directly correlated with drug resistance in SCLC cell lines. Thus, WEE1 inhibitors have the potential for synergistic interactions with various other treatment agents, underscoring their significance in combinatorial therapies for SCLC. For example, upregulation of WEE1 represents a mechanism of acquired resistance to Chk1 inhibitors in SCLC [89]. Adavosertib in combination with the PARPi olaparib enhances the efficacy of olaparib in SCLC circulating tumor cell patient-derived xenografts (CDX) [96], and a clinical trial with this combination in SCLC has been conducted under the identifier NCT02511795. Finally, WEE1 inhibition activates the STING-TBK1-IRF3 and STAT1 pathways, resulting in elevated expression of IFN-γ and PD-L1. Thus, the combination of adavosertib and PD-L1 blockade induces tumor regression, activation of type I and II interferon pathways, and infiltration of cytotoxic T cells in SCLC mouse models, representing a promising immunotherapeutic approach in SCLC [97].

Cyclin-dependent kinase 7 (CDK7) plays a pivotal role in controlling both cell cycle and gene transcription. Zhang et al. demonstrated that the selective CDK7 inhibitor YKL-5-124 induces DNA replication stress response and genome instability. Simultaneously, it activates immune response signaling, triggering a T cell-mediated surveillance system. Consistent with this, YKL-5-124 has shown improved survival in several mouse models of SCLC when combined with anti-PD-1 therapy [98]. Sun et al. showed a potential synergy between the CDK7 inhibitor THZ1 and topotecan. Inhibition of CDK7 induces RNA polymerase II proteosomal degradation. Pol II depletion prevents transcription-coupled ubiquitin-proteasome-mediated repair of topotecan-induced DNA lesions, leading to enhanced cell killing [99].

Src (c-Src) is a non-receptor tyrosine kinase that is frequently overexpressed in SCLC and plays a significant role in motility, cell adhesion, and angiogenesis. The Src inhibitor dasatinib was tested in clinical trials, showing poor results in SCLC (NCT00470054). However, the compound KC-180-2, with a dual mechanism that reduces Src phosphorylation and inhibits tubulin polymerization, was recently shown to reduce SCLC cell viability both in vitro and in vivo [100]. Additionally, YES1, a non-receptor tyrosine kinase that belongs to the Src family of kinases, has been proposed as a druggable oncogenic target in SCLC. The YES1 inhibitor CH6953755 has shown anti-tumor activity in YES1-positive organoid models as well as in cell line- and patient-derived xenografts (PDX) [101].

### 2.4. Targeting Metabolic Pathways

Heightened glucose uptake is a well-known characteristic of cancer cells, including SCLC. Consequently, research has been dedicated to investigating and targeting pathways related to glucose metabolism [102]. SCLC is distinguished by increased glutamine anabolism, which supports the synthesis of nucleic acids, thereby facilitating tumor cell proliferation. Pre-clinical studies have demonstrated that SCLC exhibits high sensitivity to the simultaneous inhibition of both salvage and de novo pathways of purine nucleotide synthesis using 6-mercaptopurine (6-MP) and methotrexate (MTX). Currently employed in clinical practice for specific types of leukemia, repurposing of this combinatory treatment for SCLC appears to be a viable prospect in the future [103]. On the other hand, SCLC tumors exhibit increased sensitivity to disruption of the pyrimidine biosynthesis pathway. Pharmacological inhibition of dihydroorotate dehydrogenase (DHODH), a crucial enzyme in this pathway, through the use of brequinar markedly reduced the viability of SCLC cells in vitro and suppressed tumor growth in vivo. This promising approach could be employed as a stand-alone agent or in combination with standard treatments for SCLC [104].

Additionally, it has been demonstrated that the MEK5/ERK5 axis regulates lipid metabolism, including the mevalonate pathway, which is responsible for cholesterol synthesis in SCLC. Depletion of MEK5/ERK5 sensitizes SCLC cells to pharmacological inhibition of the mevalonate pathway by statins, suggesting that these two kinases may be therapeutic targets in SCLC [105]. Several pre-clinical studies are in progress to investigate inhibitory molecules for these targets, such as BIX02189, which has already been studied in NSCLC but remains unexplored in SCLC [106].

In addition, Chalishazar et al. have demonstrated that different subtypes of SCLC exhibit unique metabolic vulnerabilities. The depletion of arginine using pegylated arginine deiminase (ADI-PEG20) significantly suppresses tumor growth and enhances the survival of mice, specifically in MYC-driven tumors. These findings suggest that arginine deprivation represents a subtype-specific therapeutic vulnerability [107]. Although a phase 2 clinical trial (NCT01266018) was terminated due to lack of efficacy when ADI-PEG20 was used as a single agent, an ongoing trial is currently evaluating the effects of combined treatment with gemcitabine and docetaxel in SCLC patients (NCT05616624).

In addition to its function regulating the intrinsic pathway of apoptosis, BCL2 regulates mitochondrial bioenergetics [108]. BCL2 is frequently upregulated in SCLC [109]. BCL2 inhibitors such as venetoclax [110], navitoclax (NCT03366103) [111], oblimersen sodium (G31399) [112], and palcitoclax (NCT03366103) show modest results when combined with other therapies [113], and enhance sensitivity to AURKB inhibition in pre-clinical SCLC models [114].

### 2.5. Other Targetable Proteins and Lipids Involved in SCLC

A number of cell surface proteins have been shown to play important roles in SCLC tumorigenesis. One such protein is Somatostatin receptor 2 (SSTR2), which promotes tumor growth and survival [115]. Targeting SSTR2 with the miniaturized drug conjugate PEN-221 leads to cell-cycle arrest and apoptosis, resulting in tumor regression in multiple SCLC SSTR2-expressing human xenograft models [116]. CD47 is another cell-surface molecule that acts as an anti-phagocytic signal. CD47 blockade has been shown to enhance the anti-SCLC effects of radiotherapy in murine and human pre-clinical models and to stimulate abscopal effects, inhibiting the growth of distant non-irradiated tumors [117]. There are other ADC (Antibody–Drug Conjugate) surface targets being explored in SCLC research, such as NRXN1 [118] and SEZ6 [119]. Notably, the ADC against SEZ6 ABBV-011 has advanced to a phase 1 clinical trial for relapsed SCLC (NCT03639194).

Karyopherin β1 (KPNB1) has been identified as a cytoplasmic-to-nuclear transport receptor for ASCL1 and NEUROD1 in SCLC. Inhibition of KPNB1 results in cytoplasmic accumulation and impaired transcriptional activity of both transcription factors. Pharmacologic targeting of KPNB1 using the small molecule INI-43 preferentially disrupted the growth of SCLC-A and SCLC-N cells in in vitro studies and effectively suppressed the growth of SCLC-A PDX tumors in vivo. These findings highlight the therapeutic potential of targeting KPNB1 in particular subtypes of SCLC [120].

The monosialoganglioside fucosyl-GM1 is a biomarker for SCLC [121]. BMS-986012 is a monoclonal antibody against fucosyl-GM1 that is currently being tested in a phase 1/2 SCLC clinical trial (NCT04702880). Previous studies with this antibody have shown a good tolerability profile and promising results in combination with the ICI nivolumab [122]. 

### 2.6. Targeting Pathways and Biological Processes

Developmental pathways such as Hedgehog, Notch, and Wnt are closely associated with SCLC pathogenesis. Although pre-clinical studies have demonstrated that Hedgehog inhibition can suppress tumor growth in vivo [123], a phase 2 trial conducted with vismodegib (GDC-0449) in combination with chemotherapy did not reach the OS and PFS endpoints in patients with SCLC [87]. 

Tarextumab (OMP-59R5) is a Notch2/Notch3 antagonist; in combination with platinum-etoposide, it did not improve PFS in SCLC patients (NCT01859741). Other Notch targeting strategies include inhibition of the cell-surface Notch ligand DLL3. Recently, the use of modified CAR-T cells to recognize this ligand has emerged as a personalized therapy for SCLC patients: Tarlatamab (AMG 757) has demonstrated manageable safety and response durability in patients with relapsed/refractory SCLC [124]; HPN328 and BI764532 are currently being evaluated (NCT04471727, NCT05963867). 

Wnt signaling activation has been described as a mechanism of chemoresistance in relapsed SCLC [125]. Wnt5A, a ligand of β-catenin-independent noncanonical Wnt pathways, has been identified as a promoter of SCLC neoplastic transformation and cell proliferation through activation of RHOA (Ras Homolog Family Member A). Rhosin, a selective inhibitor of GTPase activity among the RHOA subfamily, has demonstrated dose-dependent effects on SCLC viability [126]. This small molecule has been tested in various cancer animal models, exhibiting optimal safety profiles and significant efficacy [127].

Blockade of the PI3K/AKT/mTOR pathway has shown encouraging pre-clinical results, indicating a potential strategy for SCLC treatment through a combination of clinically approved PI3K and mTOR inhibitors [128]. The activation of this pathway has been described as a potential mechanism underlying therapeutic resistance [129]; thus, this approach holds promise for reversing resistance to standard therapy in SCLC. Interestingly, pharmacological inhibition of the PI3K/AKT pathway with the AKT inhibitor samotolisib delayed tumor growth and neuroendocrine transformation in an EGFR-mutant lung adenocarcinoma PDX model [130].

The MET/HGF (Hepatocyte Growth Factor) axis plays an important role in regulating cell motility in SCLC. Consequently, various MET inhibitors have been tested to target this pathway. Pre-clinical data indicate that inhibiting the MET pathway can reverse chemoresistance and impede tumor growth [131]. However, combination of MET inhibitors with chemotherapy has yielded unsatisfactory results in clinical trials [132,133]. Intriguingly, modulation of MET appears to enhance the efficacy of standard checkpoint inhibitors. Nevertheless, comprehensive pre-clinical and clinical data evaluating the combination of immunotherapy and MET inhibitors in SCLC is still missing [134].

c-Kit expression and activity are upregulated in various cancers, including SCLC [135,136]. Pre-clinical studies have demonstrated that imatinib, which inhibits c-Kit kinase activity, enhanced chemotherapy induced growth inhibition in SCLC models [137]. However, efficacy could not be demonstrated in clinical trials [138,139]. Consequently, novel strategies, such as coupling the ADC-targeting c-Kit with the microtubule inhibitor DM1, have been developed. This antibody has demonstrated significant anti-tumor activity both in vitro and in vivo and exhibits a synergistic effect when combined with carboplatin-etoposide [136].

The NFIB/CARM1 pathway plays a pivotal oncogenic role in SCLC, facilitating metastasis and regulating chromatin accessibility. Unlike NFIB, CARM1 activity can be effectively suppressed by several small molecule inhibitors that have recently become available. The CARM1 inhibitor TP-064 has been tested in SCLC PDX models, yielding the most significant reduction in tumor volume when combined with cisplatin-etoposide chemotherapy [140].

Targeting the ubiquitin-protease system (UPS) has emerged as a novel therapeutic strategy in various tumors, including SCLC. UBA1 (Ubiquitin-Like Modifier Activating Enzyme 1) is highly expressed in microcytic tumors and can be inhibited by TAK-243. SCLC cell lines and PDX models are sensitive to this agent, and it exhibits a synergistic effect when combined with chemotherapy, olaparib, and radiotherapy [141].

### 2.7. Angiogenesis Related Therapies

Angiogenesis is a mechanism that tumors often employ to sustain uncontrolled growth and avoid immune surveillance. Many tumor types benefit from therapies based on inhibition of the vasculogenesis regulator VEGF (Vascular Endothelial Growth Factor) and/or its receptor VEGFR. The use of the VEGFR inhibitor anlotinib has been approved as a third-line treatment in China, where a clinical trial demonstrated improved PFS and OS compared to placebo and a favorable safety profile [142]. Several phase 3 and phase 4 clinical trials have recently explored or are currently exploring the use of anlotinib in combination with other therapies such as chemotherapy (NCT03890055) (NCT04073550) and immunotherapy (NCT04192682). In addition, the combination of anlotinib with both chemotherapy and immunotherapy has been tested (NCT04234607). Other VEGFR inhibitors such as sunitinib and aflibercept (NCT00828139) have shown good and modest results, respectively. Pazopanib, an oral angiogenesis inhibitor targeting VEGFR, PDGFR, and c-Kit, has shown significant efficacy, prolonging PFS in extensive disease as maintenance therapy following chemotherapy [143]. Finally, the combination of the VEGFR2 inhibitor apatinib with the immunotherapeutic agent camrelizumab has demonstrated anti-tumor activity in patients who previously failed platinum-based chemotherapy [144]. Similar results for extensive-stage SCLC were achieved with the combination of the monoclonal antibody against VEGF bevacizumab and dual immunotherapy against PD-1 and CTLA-4 [145].

## 3. Conclusions and Future Directions

For decades, SCLC has been considered an undruggable cancer. However, the identification of distinct molecular subtypes, each with unique vulnerabilities, has opened a range of possibilities for repurposing or developing drugs against specific targets. Many of these agents have already been tested, and although to date there are still no approved targeted drugs for SCLC treatment, several have shown improved outcomes, especially when used in combination with chemo- or immunotherapies. In this review, we have compiled the most recent and promising pre-clinical studies (Table 1) and clinical trials (Table 2) in the field. Hopefully, with these efforts, precision medicine will revolutionize treatment for this universally lethal disease in the near future.

## Figures and Tables

**Table 1:** Targetable molecules in SCLC with positive pre-clinical results. This table does not include targets and inhibitors that have already reached clinical trials.

Target	Targeting Molecule	Results	Ref.
AURKB	Barasertib	Barasertib has growth-inhibitory effects in some SCLC lines and suppresses tumor growth xenografts. C-MYC amplification or high gene expression or C-MYC gene signature is a useful predictive biomarker.	[85]
c-Src	KC-180-2	KC-180-2 suppresses SCLC lines proliferation and inhibits the growth of SCLC xenograft tumors.	[100]
YES1	CH6953755	CH6953755 presents anti-tumor activity in organoids and in cell- and patient-derived xenografts.	[101]
HPRT1	6-MP	6-MP in combination with MTX attenuates the growth of mouse SCLC xenograft models. The glutamine synthetase inhibitor methionine sulfoximine (MSO) enhances this effect.	[103]
DHODH	Brequinar	Brequinar reduces SCLC cells viability in vitro and suppresses tumor growth in PDX and mouse models.	[104]
SSTR2	PEN-221	PEN-221 inhibits in vitro cellular proliferation and suppresses tumor growth in SSTR2-positive SCLC xenografts.	[116]
CD47	CD47-blocking antibody	CD47 blockade plus irradiation inhibits tumor growth in SCLC xenograft models, promoting abscopal effects.	[117]
NRXN1	ADC	The combination of a primary anti-NRXN1 monoclonal antibody and a secondary ADC exhibits anti-tumor activity in SCLC cell lines in vitro.	[118]
KPNB1	INI-43	INI-43 disrupts SCLC-A and SCLC-N cells proliferation in vitro and suppresses the growth of SCLC-A PDX tumors.	[120]
WNT5A/RHOA	Rhosin	Rhosin inhibits SCLC cell proliferation in vitro.	[126]
c-Kit	4C9-DM1	4C9-DM1 suppresses SCLC proliferation in vitro and tumor growth in a xenograft mouse model, with synergistic effects when combined with carboplatin-etoposide therapy.	[136]
NFIB/CARM1	TP-064	TP-064 alone cannot cause cancer regression; however, in combination with cisplatin-etoposide chemotherapy, it exhibits anti-tumor activity in a SCLC xenograft model.	[140]
UBA1	TAK-243	The combination of TAK-243 and genotoxic therapies (e.g., olaparib) exhibits a synergistic effect in cell lines and PDX models.	[141]
KDM5A/RBP2	KDM5-C70	KDM5-C70 decreases ASCL1 levels and inhibits cellular proliferation of a subset of SCLC lines. Combining KDM5-C70 and the LSD1 inhibitor ORY-1001 synergistically suppresses ASCL1 levels.	[46]
FGFR/PEA3	LY2874455	LY2874455 presents anti-tumor activity when used alone or in combination with cisplatin-etoposide in vitro and in SCLC xenografts.	[63]
STAT3 activation	Onionin A	By suppressing STAT3 activation, onionin A inhibits SCLC cells proliferation and suppresses tumor growth in a murine model.	[64]

**Table 2:** Compilation of clinical trials conducted in SCLC patients. OS: Overall survival, PSF: Progression-free survival.

Target	Targeting Molecule	Stage	Study	Results
Oncogenic transcription	Lurbinectedin	Approved	NCT04291937	Used as a second line treatment.
		Phase 3	NCT05153239	Ongoing; as a single agent or combined with irinotecan.
		Phase 3	NCT05091567	Ongoing; combined with atezolizumab.
		Phase 3	NCT02566993	Did not improve OS vs. topotecan.
		Phase 2	NCT05578326	Ongoing; combined with trilaciclib.
		Phase 2	NCT05572476	Ongoing; combined with durvalumab in pre-treated patients.
		Phase 2	NCT04607954	Ongoing; combined with durvalumab.
		Phase 1/2	NCT04358237	Ongoing; combined with pembrolizumab.
PARP	Veliparib	Phase 2	NCT02289690	Potentiates chemotherapy.
		Phase 2	NCT01638546	Improved PFS and OS in SLFN11-positive patients.
		Phase 1/2	NCT01642251	Demonstrated efficacy in patients with extensive stage SCLC when combined with chemotherapy.
	Talazoparib	Phase 2	NCT04334941	Ongoing; in SLFN11-positive patients.
		Phase 2	NCT03672773	Ongoing; combined with temozolomide in low doses.
	Olaparib	Phase 3	NCT04624204	Ongoing; in combination with pembrolizumab in pre-treated patients with chemotherapy.
		Phase 2	NCT03009682	In patients with gene mutations in the homologous recombination pathway; no results posted yet.
		Phase 2	NCT04538378	Ongoing; in combination with durvalumab in SCLC transformed from EGFR-mutated adenocarcinomas.
		Phase 2	NCT05623319	Ongoing; in combination with pembrolizumab.
		Phase 2	NCT04939662	Ongoing; in combination with bevacizumab.
		Phase 2	NCT02498613	Ongoing; in combination with cediranib in metastatic tumors.
		Phase 2	NCT05245994	Ongoing; in combination with durvalumab.
		Phase 2	NCT03428607	In combination with AZD6738; no results posted yet.
		Phase 2	NCT02937818	In combination with AZD6738 in refractory patients.
		Phase 1/2	NCT02446704	Ongoing; in recurrent patients.
		Phase 1/2	NCT05975944	Ongoing; in combination with selinexor.
		Phase 1/2	NCT02769962	Ongoing; in combination with EP0057.
		Phase 1/2	NCT02734004	Ongoing; in combination with MEDI4736.
		Phase 1/2	NCT04728230	Ongoing; in combination with carboplatin, etoposide and/or radiation.
ATR	Berzosertib	Phase 2	NCT04768296	No results posted yet.
		Phase 1/2	NCT04802174	Ongoing; in combination with lurbinectedin.
		Phase 1/2	NCT04826341	Ongoing; in combination with sacituzumab-govitecan.
	Alisertib	Phase 2	NCT06095505	Ongoing; in patients with extensive stage.
		Phase 2	NCT01045421	An objective response was noted in 21% of the patients.
Chk1	LY2606368	Phase 2	NCT02735980	Availability limitations. Another compound has been developed and is currently being tested in other neoplasms.
	SRA-737	Phase 1/2	NCT02797977	Used in combination with gemcitabine and cisplatin; no results posted yet.
WEE1	AZD1775	Phase 2	NCT02688907	Ongoing; in patients with MYC amplification or CDKN2A + TP53 mutation.
		Phase 2	NCT02593019	Used as a single agent in relapsed patients; no results posted yet.
		Phase 2	NCT02688907	Terminated, regimen change.
		Phase 2	NCT02937818	Ongoing; in combination carboplatin and olaparib.
Arginine	ADI-PEG 20	Phase 2	NCT01266018	Terminated for lack of efficacy.
		Phase 1/2	NCT05616624	Ongoing; in combination with gemcitabine and docetaxel.
BCR-ABL, PDGFR, c-Kit	Imatinib	Phase 2	NCT00156286	No results posted.
		Phase 2	NCT00248482	No results posted.
		Phase 2	NCT00052949	No results posted.
		Phase 2	NCT00193349	No results posted.
EZH2	DS-3201b	Phase 1/2	NCT03879798	In combination with irinotecan; no results posted.
	XNW5004	Phase 1/2	NCT06022757	Ongoing; in combination with pembrolizumab.
VEGFR, PDGFR and c-Kit	Pazopanib	Phase 2	NCT01253369	Notable rate of stable disease and slightly improve of PFS compared to second-line therapies.
BCL2	Oblimersen Sodium	Phase 2	NCT00042978	No results posted yet.
GM1	BMS-986012	Phase 1/2	NCT04702880	Ongoing; in combination with carboplatin, etoposide and nivolumab.
		Phase 1/2	NCT02247349	No results posted yet.
LSD1	Iadademstat	Phase 2	NCT05420636	Ongoing; in combination with paclitaxel in relapsed patients.
DLL3	AMG 757	Phase 3	NCT05740566	Ongoing; in combination with chemotherapy.
		Phase 2	NCT05060016	Ongoing; in relapsed patients.
	HPN328	Phase 1/2	NCT04471727	Ongoing; in patients with DDL3 expression.
		Phase 1/2	NCT05879978	Ongoing; in combination with ezabenlimab in patients with DDL3 expression.
	BI764532	Phase 2	NCT05882058	Ongoing; different doses of the compound tested.
CREBBP	Tinostamustine	Phase 1/2	NCT03345485	No results posted yet.
VEFGR	Sunitinib	Phase 2	NCT00695292	No OS and PFS results posted.
		Phase 2	NCT00620347	Approved as a second-line treatment in China.
		Phase 2	NCT00616109	No improvements over control.
		Phase 2	NCT01306045	Ongoing; tested among several drugs.
		Phase 1/2	NCT00453154	Safe and improved PFS.
	Anlotinib	Phase 4	NCT03890055	In combination with chemotherapy in relapsed patients.
		Phase 3	NCT04073550	In combination with topotecan.
		Phase 3	NCT04234607	In combination with benmelstobart, etoposide and carboplatin. No results posted yet.
		Phase 3	NCT04192682	In combination with sintilimab.
	Aflibercept	Phase 2	NCT00828139	In combination with topotecan. Improved PFS but increased toxicity.
VEGF	Bevacizumab	Phase 2/3	NCT00930891	In combination with chemotherapy, did not improve outcome in extensive SCLC patients.
		Phase 2	NCT00403403	In combination with cisplatin or carboplatin plus etoposide for treatment of extensive-stage SCLC, improved PFS with an acceptable toxicity profile. No improvement in OS was observed.
		Phase 2	NCT04730999	Ongoing; in patients with extensive disease.
		Phase 2	NCT04939662	Ongoing; in combination with olaparib.
		Phase 2	NCT00698516	After treatment with topotecan-bevacizumab, improvement in PFS.
		Phase 2	NCT05588388	Ongoing; in combination with chemo-immunotherapy and atezolizumab.
		Phase 2	NCT00193375	2-Year PFS: 22% of participants. Overall response rate: 80%.
		Phase 2	NCT00726986	Terminated due to extreme toxicity when combined with cisplatin and etoposide.
VEGFR2	Apatinib	Phase 3	NCT03651219	No results posted.
		Phase 3	NCT04490421	No results posted.
		Phase 3	NCT02875457	No results posted.
		Phase 3	NCT03100955	No results posted.
		Phase 2	NCT03547804	Successful, already in Phase 3.
		Phase 2	NCT04901754	In combination with camrelizumab; no results posted yet.
		Phase 2	NCT04128800	In combination with S-1; no results posted yet.
		Phase 2	NCT04683198	Ongoing; in combination with cambralizumab, carboplatin and etoposide.
		Phase 2	NCT02945852	No results posted.
		Phase 2	NCT02995187	No results posted.
		Phase 2	NCT03135977	No results posted.
		Phase 2	NCT03389087	No results posted.
		Phase 2	NCT02980809	No results posted.
		Phase 2	NCT03129698	No results posted.
		Phase 2	NCT03417895	No results posted.
		Phase 2	NCT04659785	No results posted.
		Phase 2	NCT04453930	No results posted.

## Data Availability

Not applicable.
